# Human–Robot Interface for Embedding Sliding Adjustable Autonomy Methods

**DOI:** 10.3390/s20205960

**Published:** 2020-10-21

**Authors:** Piatan Sfair Palar, Vinícius de Vargas Terres, André Schneider de Oliveira

**Affiliations:** Graduate School of Electrical Engineering and Computer Science (CPGEI), Federal University of Technology-Paraná (UTFPR), Avenida 7 de Setembro 3165, Curitiba 80230-901, Brazil; viniciusterres@alunos.utfpr.edu.br (V.d.V.T.); andreoliveira@utfpr.edu.br (A.S.d.O.)

**Keywords:** sliding autonomy, human–robot interface, Myo armband, fuzzy controller

## Abstract

This work discusses a novel human–robot interface for a climbing robot for inspecting weld beads in storage tanks in the petrochemical industry. The approach aims to adapt robot autonomy in terms of the operator’s experience, where a remote industrial joystick works in conjunction with an electromyographic armband as inputs. This armband is worn on the forearm and can detect gestures from the operator and rotation angles from the arm. Information from the industrial joystick and the armband are used to control the robot via a Fuzzy controller. The controller works with sliding autonomy (using as inputs data from the angular velocity of the industrial controller, electromyography reading, weld bead position in the storage tank, and rotation angles executed by the operator’s arm) to generate a system capable of recognition of the operator’s skill and correction of mistakes from the operator in operating time. The output from the Fuzzy controller is the level of autonomy to be used by the robot. The levels implemented are Manual (operator controls the angular and linear velocities of the robot); Shared (speeds are shared between the operator and the autonomous system); Supervisory (robot controls the angular velocity to stay in the weld bead, and the operator controls the linear velocity); Autonomous (the operator defines endpoint and the robot controls both linear and angular velocities). These autonomy levels, along with the proposed sliding autonomy, are then analyzed through robot experiments in a simulated environment, showing each of these modes’ purposes. The proposed approach is evaluated in virtual industrial scenarios through real distinct operators.

## 1. Introduction

Robots have been a reality in human lives for decades and facilitate societies in several repetitive, tedious, and dangerous tasks. Industrial duties, such as casting, stacking, welding, sorting, and coating have been performed for more than 30 years globally [1]. With technological advancement, increased communication and cooperation between robots and human operators are necessary throughout, which is called human–robot interaction (HRI). This interaction has evolved from buttons and switches [2] and increasingly resembles natural human communication, combining the best competencies of both humans and robots, closing in previously distant agents, and removing the need for more operators, along with lengthy and costly training drills [3]. Some areas where HRI is more prevalent include education, medical school, elderly care, military, space exploration, disaster situations, and industries. The latter is the focus of this work—in particular the prevalence of HRI in Industrial Inspection.

HRI, according to Goodrich and Schultz [4], can be defined as the field of study comprising conception to final development, of robots that interact with humans physically, emotionally, or socially. Industrial robots, robotic arms, autonomous cars, assistant robots, and rescue robots are some examples of robots that can collaborate, communicate, or cooperate with humans [5].

In industry, HRI can be observed in processes such as robotic arm movements in collaboration with assembly processes, automatic orientation with crane robots, and cooperation in riveting processes, to name a few. Technologies, such as human and object detection in 3D environments and gesture recognition, are generally utilized to accomplish these tasks [6].

In disaster recovery and search-and-rescue situations, for example, robots can be deployed in dangerous places while being teleoperated by an operator from a safe station. This distance can lead to some challenges for safe operation, such as communication delays and difficulties in monitoring and analyzing the situation [7]. Subsequently, a new research field emerged, which defined the rules for the autonomy levels that robots assume in specific tasks and in emergency situations or anomalous operations.

In dynamic real world applications, where ever-increasing automation is pursued, there are countless variables and unexpected errors that can occur while conducting tasks. Robots can be reprogrammed each time a new situation is detected or a qualified human operator who can address the problem can be reached. In contrast, human operators also have constraints, and if compelled to work for extended periods, can face exhaustion that can undermine their attention span and decision-making ability. As both agents have limitations and complementary skills, it is critical that a union between both agents is formed to perform tasks in a more efficient and safe manner, with a method to select which agent accounts to what extent for each variable and at which time.

This paper presents the development of an interface that enables a specialized operator to control an inspection robot throughout its tasks in storage tanks. This task can be unwrapped into several other tasks, each with its own unique requirements related to different autonomy levels. By performing nondestructive testing with phased array ultrasonic sensors, the operator might need to walk back from a route, slow down in some segments, or carry out the entire inspection at once. Different operators with different skill levels could also require more or less aid while performing inspection tasks. This work aims to present a method that employs sliding autonomy techniques to assist operators in any case/situation during an inspection, thus providing more freedom so that the operator can focus on the inspection regardless of his/her skill level or difficulty of the task.

In Section 2, an overview of the levels of autonomy in robotics is presented. Section 3 describes the implementation of the HRI along with the sensor fusion. In Section 4, simulation experiments are presented and discussed, and in Section 5, the conclusions are drawn.

## 2. Overview of Levels of Autonomy in Robotics

One of the most complete definitions for autonomy, considering the social aspect of an agent, is the notion that an agent is autonomous when they have the ability to choose to act in a way that goes against the choices of the other agents [8]. An agent might then possess multiple levels of autonomy, established by the extent to which their actions are affected by the other agents. Another scope for autonomy in robotics is how long a robot can be ignored. The more time a robot can operate without human interruption, the greater is their autonomy level [4]. Safety basics in autonomy levels are a topic of study in specialized papers such as Lasota et al. [5] and Çürüklü et al. [9].

In several implementations, some mechanism is required to change between the levels of autonomy. A robot performing fully autonomous tasks might need assistance in some situations, or different tasks could require various levels of autonomy. While the fully autonomous level exempts the attention of the operator, it can lead to undesirable scenarios and even jeopardize the safety of humans and machines. In contrast, when an operator takes over and controls every task, a greater safety in the meaning of the task is achieved with potential efficiency loss [10]. Therefore, varying the level of autonomy in intelligent systems is a compromise between convenience and safety. In the robotics setting, the work presented by Desai and Yanco [11] separates the levels of autonomy into four modes, with the two extremes being teleoperation and autonomous. The intermediary levels are safe, where the operator controls the robot, which can avoid unsafe operation and collisions, and shared, where the robot is autonomous but allows the user to intervene in its movements. When these levels are altered during operation, sliding autonomy is achieved. Other terms to describe this operation are adjustable autonomy and mixed initiative. In Hardin and Goodrich [12]’ work, there is a separation between the above-mentioned terms: adaptive autonomy is the variation of autonomy only by the robot itself; adjustable autonomy refers the variation made by an operator only; mixed initiative involves a collaboration between the robot and operator to find the best level of autonomy for the system. In the same study, simulation experiments were performed to determine which of these three methods would be more effective in a search-and-rescue scenario with 200 robots searching for five people placed randomly in a map. The conclusion was that, with a procedure simulating previous knowledge from the operator, a better result could be achieved with both the operator and robot defining the goals and autonomy levels.

In Desai and Yanco [11], the researchers defined parameters to better portray the levels of autonomy of an autonomous robot navigating with obstacles. By modifying the values of these parameters, countless levels of autonomy can be achieved. The parameters defined in the study included inflated obstacle distance, user-defined speed, robot-defined speed, split of the speed contribution by the agent, speed limits, and deviation of obstacle distance. Several experiments were performed while shutting down and changing some of the parameters to achieve different results. The authors reported that all these parameters were defined by the user, and suggested additional research on how the robot could by itself change these values and modify the level of autonomy. One option would be to utilize artificial intelligence techniques to estimate these parameters in a safe and autonomous manner. This could help in instances where the robot needs assistance. Instead of the entire task being performed solely by an operator, the autonomy levels could be lowered so that the operator can help the robot until it realizes that human aid is no longer necessary, and subsequently increase the autonomy level back to autonomous again. In another similar paper [13], sliding autonomy was achieved through three levels, namely teleoperation, autonomous, and intermediate, through destiny points and heuristics. In this mode, general direction information was inserted in advance, defining places in the map for the robot to follow and those to avoid. The final speed was achieved by combining the information from the map and the current goal of the robot.

A research study [14] was conducted where the autonomy level was tailored to match the experience and skill of the operator. First, the skill of the operator was tested and measured in three experiments, to ascertain the navigation skills, handling, and cooperation. The results were then considered to define the level of autonomy in the final experiment, which involved two robots that were required to carry objects together. In the experiments, the system performance achieved with adapted autonomy and fixed autonomy was compared, and the results suggested the requirement of less time for performing the task with fewer falls using adapted autonomy.

It has been observed that the approaches concerning the autonomy levels vary according to the field of application, following the robotics pattern between three to five different levels of autonomy. Some applications have a pre-established level of autonomy, called offline, which does not change during operation. In other applications, the level of autonomy should be changed during the operation time, which is called online.

A summary of references grouped according to the field of application is presented in Table 1. The second column indicates the number of autonomy levels used in the corresponding fields and, with the subsequent columns indicating whether these levels are changed in an online or offline manner.

In search and rescue robotics, while a predetermined autonomy level exists for the robots, this level can be changed during operation if aid is requested by the robot. This trend is also observed in the military field, where the level of autonomy is pre-established, but in some specific situations, authorization from an operator or even a change in the autonomy level is required to accomplish certain tasks such as when defusing explosives. Another common point that should be noted in military applications, natural disasters, and space robots is that, as operators are far away from the robots due to hazardous activities, communications failure should be considered, and the robots should change their autonomy levels to prevent accidents.

In mobile robotics, a majority of studies present an offline change in the autonomy levels. In some cases, a middle ground between the operator and robot controls is used. In other cases, experiments are first conducted to assess the skill or experience of the operator and then the level of autonomy is set.

This work’s main contribution in relation to other solutions with sliding autonomy is that this approach constitutes an automatic method of selecting the level of autonomy and changing it during operation—i.e., it is adaptive. This means that the user does not select a level of autonomy before the task and not even during the task. Still, the system is sensing the inputs and defining the level of autonomy to any situation during operation. Another contribution of this study is that the system is acting to help correct the operator’s inputs while giving the operator the impression of control. The system increases autonomy only if the operator’s actions are jeopardizing the task.

In the following section, the implemented autonomy levels are presented, along with the method used to achieve sliding autonomy.

## 3. Implementation of the Human-Robot Interaction

In this section, the interface between the operator and robot is presented. The task that needs to be completed, the robot, the input devices, and the controller used to change the autonomy levels in this study are also presented in this section.

### 3.1. Tank Inspection Task

**Figure 1:**
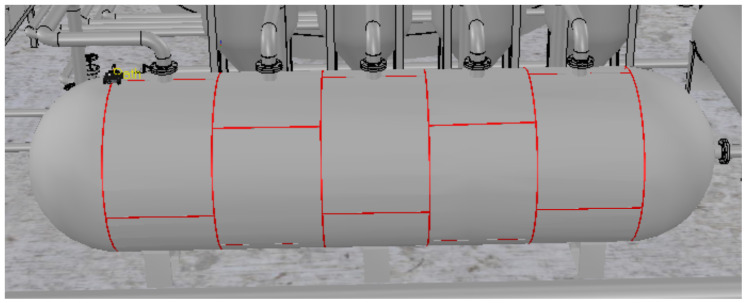
Storage tank in a refinery. The weld beads are highlighted in red.

A storage tank containing liquefied petroleum gas is shown in Figure 1. In this figure, the weld beads that unite the metal plates are highlighted in red.

To accomplish the external inspection task, ultrasonic sensors are brought near the weld beads and then all the bead lengths are covered while the operator checks for weld failures that can cause problems in the tank structure using the inspection device. For a robot to execute this task, it must first be brought close to the tank until magnetic attachment is achieved. Then, the robot has to find the weld bead and cover all the bead lengths while keeping the bead centered on the inspection sensor. Some difficulties that can occur during inspection can be due to factors, such as magnetic attachment during all tank sectors, including upside down and vertical climbing where gravity plays a role; pipes, obstacles, and imperfections on the surface of the tank that can obstruct the programmed route or inflict loss of the weld bead or even magnetic attachment; the assessment of intersections and performance on turns, which can hinder the localization of the route to be covered by the weld beads.

### 3.2. Autonomous Inspection Robot

**Figure 2:**
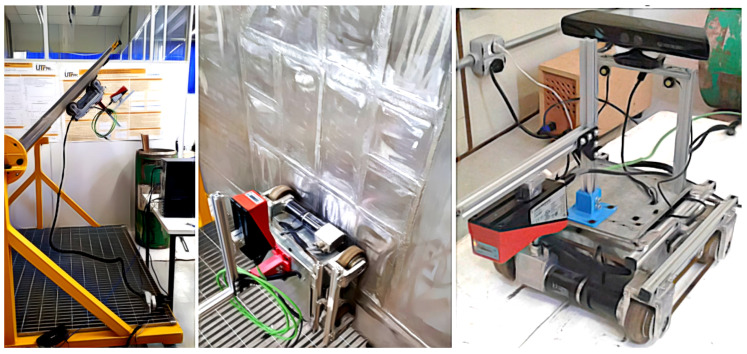
Autonomous Inspection Robot 1 (AIR1). A robot conceived/developed to inspect weld beads in storage tanks containing liquefied petroleum gas. A profile sensor, facing downwards, is positioned in front of the robot.

The autonomous inspection robot 1 (AIR1), shown in Figure 2, was developed to inspect weld beads in storage tanks and spherical pressure vessels containing liquefied petroleum gas (LPG). AIR1 was developed at Universidade Tecnológica Federal do Paraná (UTFPR) in the LASCA laboratory, by [30,31,32].

The storage tanks and pressure vessels inspected by AIR1 operate at temperatures close to 100 degrees Celsius. Two brushless motors move four magnetic wheels through a differential system, where turning is achieved via angular speed variation between the left and right wheels. Wheels on the same side are connected by a toothed belt, and all the wheels are magnetic because the robot needs to move horizontally, vertically, and even upside down to climb and fully inspect the tanks.

**Figure 3:**
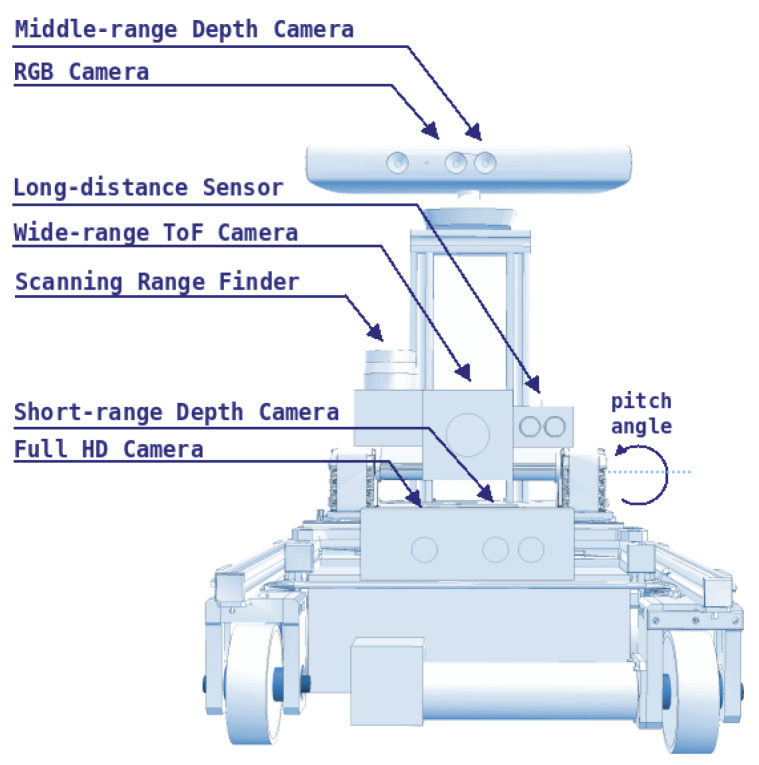
Sensors for perception of the environment previously attached to AIR1. Depth cameras and Lidar sensors were used to map the tanks and to predict the sizes of spherical pressure vessels (adapted from [34]).

AIR1 has been used in previous research with several sensors for different purposes. For a safe operation, Teixeira [33] developed a system able to sense and estimate the environment of tanks and pressure vessels with the aid of Lidar sensors and depth cameras that can be seen in Figure 3. These sensors can also detect pipes and other structures around the robot to prevent collisions. However, since there is no interest in mapping the tanks or avoiding obstacles, the cameras and Lidar sensors were taken out, giving a profile sensor to measure the weld bead. This modular approach to AIR1, where sensors can be replaced according to the current research focus, has helped create several solution, while the final architecture of AIR1 is still in development.

### 3.3. Input Devices

An industrial joystick, usually employed to control cranes and lift trucks, was designed for this work. It consists of a transmitter and receiver, communicating through radio frequency. The transmitter consists of two analog sticks with two speeds each, two on/off buttons, and four push buttons. An emergency button is located on the controller’s side and can stop the robot if the operator perceives any unsafe movement. The joystick is ISO 13849-1 compliant with a level of performance ”d”, indicating a probability of dangerous failure per hour within the 0.00001% to 0.0001% range. The joystick also carries an IP65 enclosure, being “dust tight” and protected against water projected from a nozzle, increasing the safety of the operation.

A driver was developed to communicate between the receiver and robot, reading the signals from the relays of the receiver and, with the aid of an Arduino Due, communicating with the robot. The developed software converts the signals from the receiver into a standard ROS message, defined as a sensor_msgs/Joy message. The advantage of using standard messages is that this joystick and driver can be used in other robotic applications, as they exchange information with ROS through a standard message. The communication between Arduino and the computer controlling the robot is established through serial communication using the rosserial_arduino package, available at http://wiki.ros.org/rosserial_arduino.

The data of the generated sensor_msgs/Joy message are then analyzed in a created C++ ROS node. This node reads the information from the /joy topic, which is converted into speed velocities published in the /joy/cmd_vel topic.

Through the joystick, the operator acts with angular and linear velocities. This information from the transmitter is transmitted via radio frequency to the receiver, and through the driver developed in Arduino, to the computer connected to the robot, which then moves.

One difficulty encountered using the joystick to control the robot and to detect the user’s intention was the limited information of the joystick’s analog sticks that can only provide two speeds. The price of a fully analog stick with a higher sensitivity with the industrial robustness required for this application was out of reach for this project. To obtain more information about the intent of the user, an electromyographic armband called Myo was added to the system. The Myo armband has been extensively used in robotic studies. This armband holds eight electrodes that can perform surface electromyography in the arms and, through mathematical analysis, detect some standard hand signals or gestures made by a person, such as the rest position, closed fist, hand palm outside or inside, separated fingers, and thumb to little finger. This armband also contains an inertial measurement unit (IMU) that can measure the three Euler angles [35].

Some studies with the Myo armband in the robotics context are discussed below. In two similar papers, presented by Tortora et al. [36] and Xu et al. [37], the authors used two armbands, one in the arm and the other in the forearm to, via their inertial sensors, reconstruct the operator’s arm movement in the teleoperated robotic arms. Furthermore, in the work of Kim et al. [38], the arm movements were reconstructed using only one armband in the upper part of the arm. The forearm movement was calculated using electromyographic sensors. Another similar work, presented by Çoban and Gelen [39], used the armband to remotely operate a robotic arm through its inertial sensors. In this study, gesture detection was also added to accomplish certain tasks, such as the opening and closing of a claw manipulator. Another robotic arm, presented in Pambudi et al. [40], was controlled via standard gestures and an artificial neural network. Gestures were also used in the work [41] to control the direction of movement of a mobile robot, while the velocities were controlled by the arm’s stiffness. This paper also utilizes the armband’s vibration function to provide haptic feedback to the operator.

**Figure 4:**
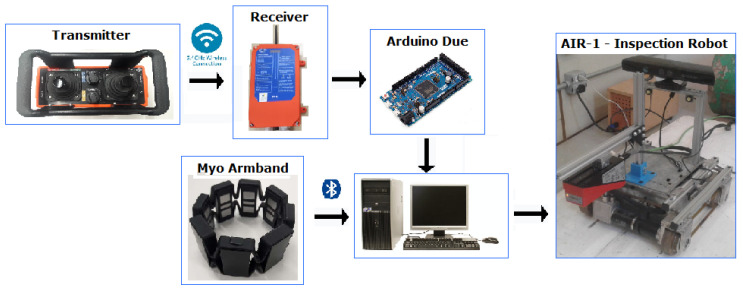
System architecture. An industrial joystick is operated by sending signals via radio frequency to a receiver. The data are processed in the Arduino DUE and sent to the computer that controls the robot. The operator also wears a Myo armband to assist with the robotic control.

**Figure 5:**
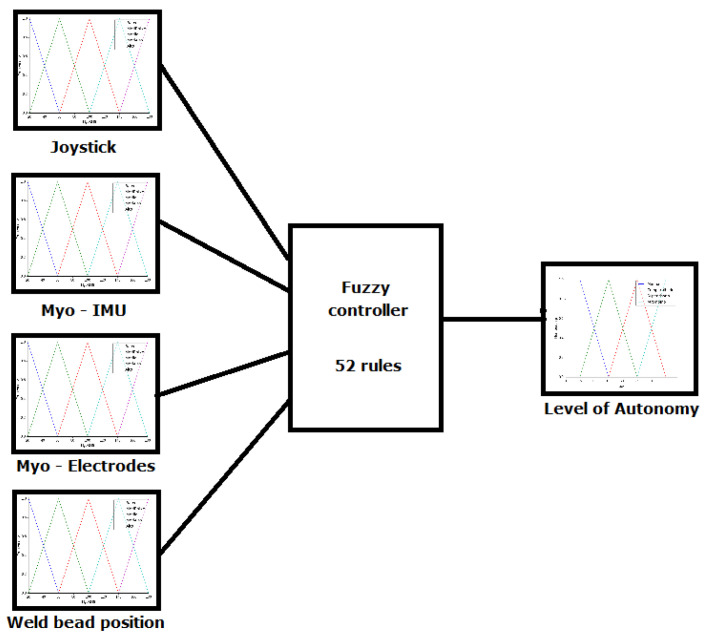
Diagram of the Fuzzy controller implemented.

**Figure 6:**
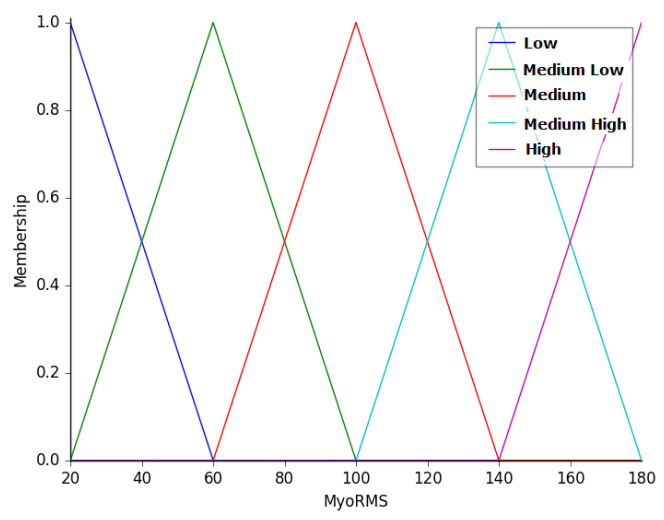
Membership functions of the variable MyoRMS.

**Figure 7:**
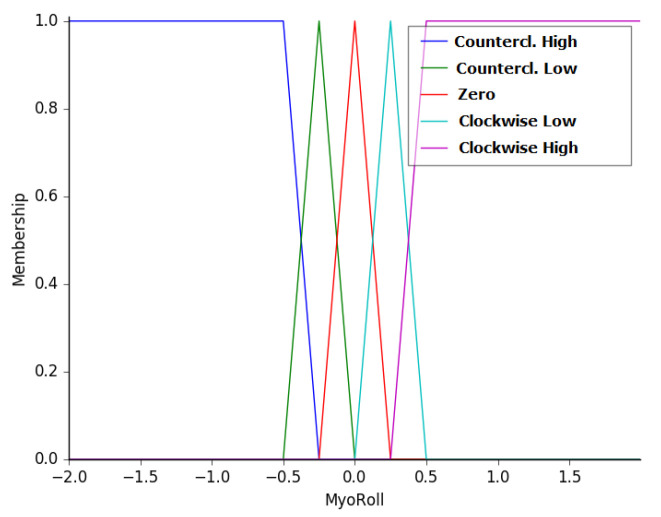
Membership functions of the variable MyoRoll.

**Figure 8:**
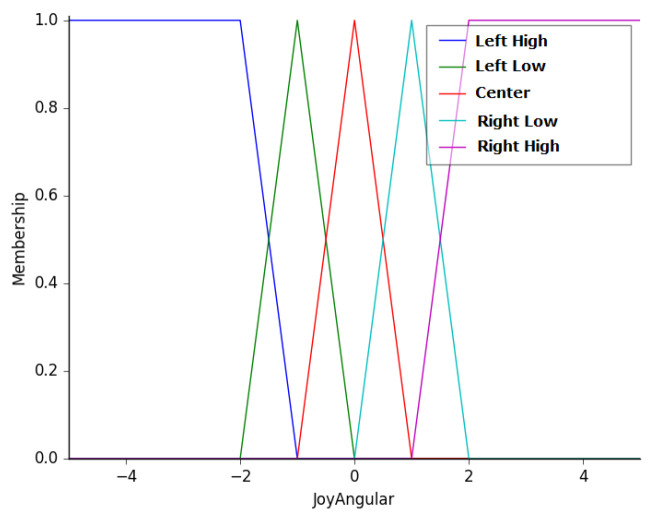
Membership functions of the variable JoyAngular.

**Figure 9:**
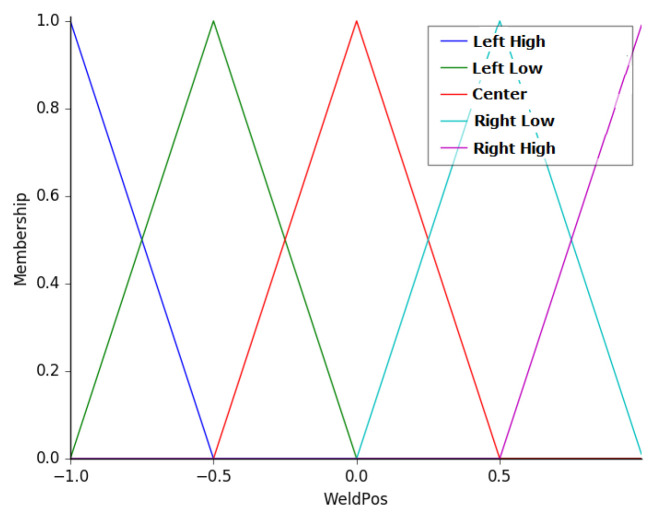
Membership functions of the variable WeldPos.

In addition, research studies have used the the raw data of the electromyographic sensors to detect other gestures apart from the standard ones. In the work of Krishnan et al. [35], five gestures of the hand cricket game were detected using machine learning techniques, such as the support vector machine. In the work of Luh et al. [42], 16 finger gestures were categorized with the aid of artificial neural networks. The research study reported by Kurniawan and Pamungkas [43] also categorized two gestures through artificial neural networks. In the work presented by Li et al. [44], five gestures were classified using the K-nearest neighbors classifier to move an omnidirectional mobile robot. In Kaya and Kumbasar [45], 10 hand gestures representing the figures 0–9 were classified by the authors using different machine learning algorithms, namely the K-nearest neighbors, support vector machine, and artificial neural networks. Another extensive research study [46] used transfer learning to classify 7 and 17 different gestures. In the study presented by Wijayasinghe et al. [47], the inputs of the Myo armband were mapped linked to the robot movement using artificial neural networks and genetic algorithms. In this case, the error function was created considering the duration of the experiment.

In another area of research in the robotics field, the Myo armband is used in combination with other input sources to control the robots. In the work of Chen et al. [48], hand signals were used along with voice commands to control an omnidirectional mobile robot equipped with ultrasonic sensors to avoid collisions. In the research presented by Kunapipat et al. [49], the Myo armband was used together with a 5DT data glove to identify 10 arm movements.

In another study [50], the performance drop of the armband in people with muscle injury was demonstrated. This study compared healthy people with those with muscle injury after 10, 40, and 80 s of performing gestures to move the slides in a presentation.

Using the ros_myo package, available at https://github.com/uts-magic-lab/ros_myo, the data from the Myo armband can be obtained via Bluetooth in the ROS standards. The ros_myo package provides a ready-made solution delivering eight types of information in the format of ROS topics ready to be published or subscribed to. In this study, four of those topics were used and consisted of the following:

- /myo_raw/myo_emg: This topic contains a vector with the raw data from eight electrodes;
- /myo_raw/myo_ori: Contains the value, in radians, of the roll, pitch, and yaw rotation angles from the IMU;
- /myo_raw/myo_gest_str: This string contains the name of the gesture being performed by the user. These gestures are standardized and are comprised of six hand positions: rest, fist, wave in, wave out, fingers spread and thumb to pinky;
- /myo_raw/vibrate: Contains a value ranging from 0 to 3. A value of 0 corresponds to no vibration, 1 is weak vibration, 2 is medium vibration, and 3 indicates strong vibration providing haptic feedback to the user.

A summary of all the input devices used/included in the system is presented in Figure 4.

### 3.4. Autonomy Levels and Sliding Autonomy

The levels of autonomy in the robotic field are usually divided into four modes, namely manual, shared, supervisory, and autonomous [11]. These levels were implemented in this study to not only provide comfort and safety in the operation, but to also provide total control to the operator if needed. An intelligent method of selection for the best level of autonomy was also developed and will be explained in this section.

To accomplish the change in the level of autonomy during operation, which is called sliding autonomy, a fuzzy controller was developed. The fuzzy controller was selected because there is no mathematical model of the autonomy selection system, and this type of controller allows for the analyses of the system in an intuitive manner. A Mamdami type controller was developed, with the implication operator set as the minimum, aggregation operator set as the maximum, centroid as the defuzzification method, and four inputs and one output, as illustrated by the diagram of this controller in Figure 5. Other types of controllers that have been used to select autonomy levels in the literature are the Markov decision process [16,51], finite state machine [27], and genetic algorithms and artificial neural networks [47].

Figure 5 shows the four input variables, namely the angular velocity input on the joystick, Euler angles detected by the IMU, value of the electrodes in the armband, and the weld bead position relative to the center of the robot. The output variable is the level of autonomy of the robot.

This controller has the goal of detecting the level of skill of the operator and adjusting the appropriate level of autonomy, resulting in a smooth operation. Through haptic communication, the armband gives feedback to the user, with increased vibration when the user provides incorrect inputs.

The first input of the controller is the root mean square (RMS) value measured by the electrodes of the armband in a window containing the 50 most recent readings. This value represents the movement of the forearm or its stiffness. The forearm’s stiffness increases if the user is nervous or making large and wide movements, indicating uncertainty in his movements. On the other hand, if the user is calm and performing small and smooth forearm movements, this variable will have a lower value, indicating that the user has more certainty in controlling the robot. This variable was termed as MyoRMS and the linguistic variables associated with this entry are low (LW), medium low (ML), medium (MD), medium high (MH), and high (HG). Figure 6 shows the membership functions of this linguistic variable, whose universe ranges from 20 to 180.

The second input is the value of the difference in the rotation angle detected by the IMU of the Myo armband. This angle increases when the arm rotates clockwise, mimicking the movement of tightening a screw or inserting a light bulb. This value facilitates smoother robot movement because the analog sticks in the joystick only represent two velocities. This variable was termed as MyoRoll and the membership functions associated with this variable are as follows: counterclockwise high (NH), counterclockwise low (NL), zero (ZR), clockwise low (CL), and clockwise high (CH). These functions, ranging from −2 to 2, are presented in Figure 7.

The third input is the value of the angular velocity input/selected by the operator on the joystick. As mentioned previously, as the analog sticks of the joystick can only handle two speeds on each side, the value is always 0%, 50%, or 100% of the specified value. To ease this situation, buttons on the sides of the joystick can be pressed to adjust, upwards or downward, the specified value of the angular and linear velocities. This variable was termed as JoyAngular, and its membership functions are left high (LH), left low (LL), center (CT), right low (RL), and right high (RH). Figure 8 presents the membership functions of this variable, which ranges from −5 to 5 rad/s.

The fourth input is the distance between the weld bead position and the center of the robot. This variable was named as WeldPos, and the membership functions associated with this variable are: left high (LH), left low (LL), center (CT), right low (RL), and right high (RH). Figure 9 presents the membership functions of this variable, which universe ranges from −1 to 1, representing the width of the profile sensor.

**Figure 10:**
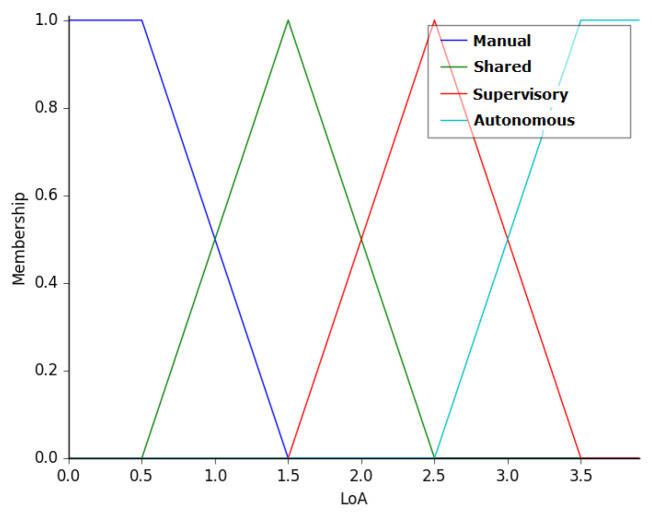
Membership functions of the variable LoA—i.e., level of autonomy.

The output of the fuzzy controller is the level of autonomy to be employed by the robot. This variable was termed as LoA, i.e., level of autonomy. The membership functions of this variable are manual (MN), shared (SH), supervisory (SP), and autonomous (AT), as shown in Figure 10. This variable ranges from 0 to 4.

The inputs of the controller are related to the output via fuzzy rules, as presented in the tables. Table 2 shows the relationship between the angular velocity on the joystick with the weld bead position relative to the center of the robot. The more aligned these values are, the lower is the output level of autonomy of the system.

Table 3 presents the relationship between the rotation of the arm measured by the IMU and the weld position relative to the center of the robot. Similarly, when the values are aligned, is the robot has less autonomy.

The last rule set is presented in Table 4. The relationship shown in this table represents the level of autonomy changed by the RMS value of the electrodes of the armband. After testing, only the medium high and high levels of MyoRMS are considered, indicating some situations where the operator is under high pressure or experiences uncertainty in their movements.

Next, the levels of autonomy implemented are presented.

#### 3.4.1. Manual Mode

**Figure 11:**
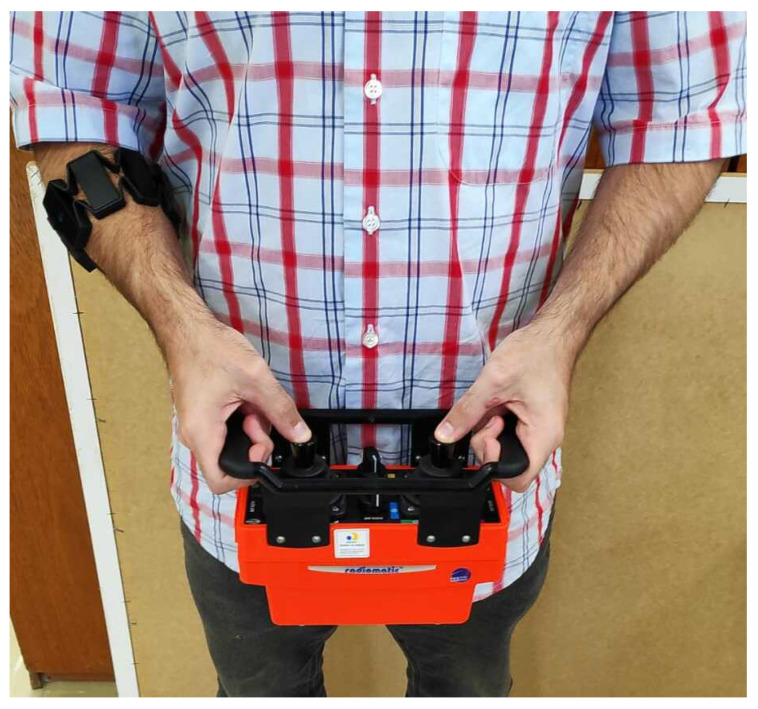
Robot controls. There is a fusion between data from an industrial joystick and a Myo armband to control the robot.

When the output of the fuzzy controller is in the 0–1 range, the level of autonomy of the robot is set to manual mode. In this mode, the operator has full control of the inspection robot and can freely move the robot on the tank or surface to be inspected. This is the standard mode for positioning the robot in the tank or in a weld bead, to detach the robot from the tank or to re-position the robot when the weld bead is lost by the profile sensor. To move the robot, the operator controls the joystick in combination with the rotation of the arm to generate a smoother movement to the robot, which is presented in Figure 11.

#### 3.4.2. Shared Mode

When the output of the fuzzy controller is in the 1–2 range, the level of autonomy is set to shared mode. In this mode, the operator and robot act together to control the robot. The mixed initiative can be achieved with the operator acting similarly in manual mode and the robot via the autonomous mode presented in Section 3.4.4. Each agent can contribute 0 to 100% of the final velocities, where one agent complements the other. The final velocity commands sent to the robot are calculated using Equation (1).

(1) FinalVelocity=(LoA−1)×AutonomousVelocity+(2−LoA)×ManualVelocity

**Figure 12:**
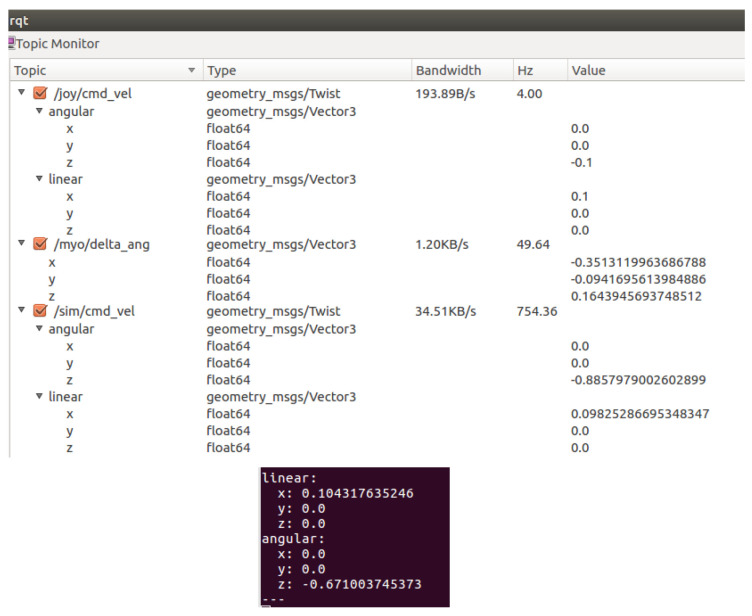
The velocities of the robot are published in ROS topics.

In the case where the output of the fuzzy controller is 1.7, for example, the velocity input of the operator comprises 30% of the final value, with the autonomous system responsible for the remaining 70%. The velocities can be seen in the ROS topics, as shown in Figure 12. The topic /joy/cmd_vel shows the velocities entered by the operator on the joystick while the topic /sim/cmd_vel shows the velocities that the autonomous system is indicating. The final velocity of the robot is found in another topic, displayed in the rectangle at the bottom of the figure.

#### 3.4.3. Supervisory Mode

If the output of the fuzzy controller is in the 2–3 range, the level of autonomy is set to the supervisory mode. In this mode, the angular velocity of the robot is autonomous, while its linear velocity is manual. In this way, the robot does not lose sight of the weld bead and the operator has full control of the linear velocity, with the robot moving forward or backward at the appropriate speed.

#### 3.4.4. Autonomous Mode

**Figure 13:**
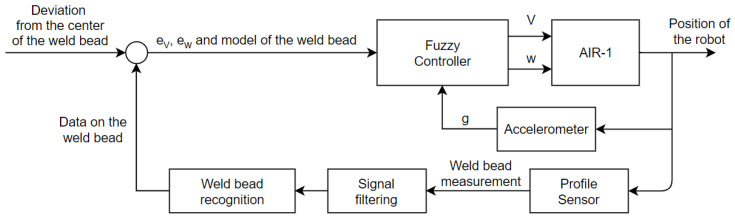
Block diagram of the autonomous mode. The control system reported by Terres [52] was adopted. A fuzzy controller controls the robot in an autonomous way to follow the previously identified weld bead.

The last level of autonomy is achieved when the output of the fuzzy controller is in the 3–4 range. This mode is called autonomous and consists of a separate fuzzy controller, as presented by Terres [52]. In that study, a communication interface with a profile sensor was developed to read the data from the sensor in a standard sensor_msgs/LaserScan ROS message. A weld bead identification system was also developed by Terres [52] along with a weld bead follower, which was adapted in this work as the autonomous mode. A block diagram of the developed weld bead follower is presented in Figure 13.

The other fuzzy controller reported by Terres [52] contains four inputs and two outputs. The inputs are: (1) the distance between the center of the alignment of the robot and the center of the weld bead, (2) the orientation error of the robot, (3) the measurement model of the weld beads, and (4) gravity. The outputs are the linear and angular velocities of the robot. A Python script executing the above logic was partially altered in this work, wherein the joystick inputs were added to control the direction of robot movement at intersections. This modification was implemented because in the original work, this decision was random. Another addition is the possibility of the robot following a previously traced route that can be expanded in trajectory computations in the autonomous mode.

#### 3.4.5. Application Examples

**Figure 14:**
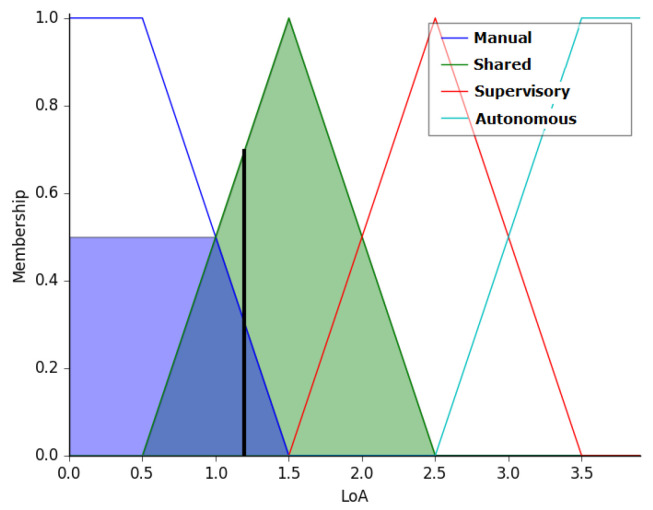
Output of the Fuzzy controller for the input vector (50, −1.5, −0.5 −1). The output is Shared Mode, with 81% of the final speed being controlled by the operator and 19% by the autonomous system.

As an example, we consider the following inputs to the controller: MyoRMS value at 50, depicting low arm stiffness; JoyAngular value of −1.5, indicating a low left movement; WeldPos value of −0.5, indicating that the weld position is more to the left; MyoRoll at −1, indicating a counterclockwise low movement. This input vector represents an operator with a correct movement but with a small excess in the rotation of the arm. The activated rules tend to output the level of autonomy more on the manual side. When this input vector is fed to the fuzzy controller, the output is 1.19, indicating operation in shared mode with 81% of the final speed of the robot being controlled by the operator and 19% by the autonomous system. Figure 14 illustrates the above example.

**Figure 15:**
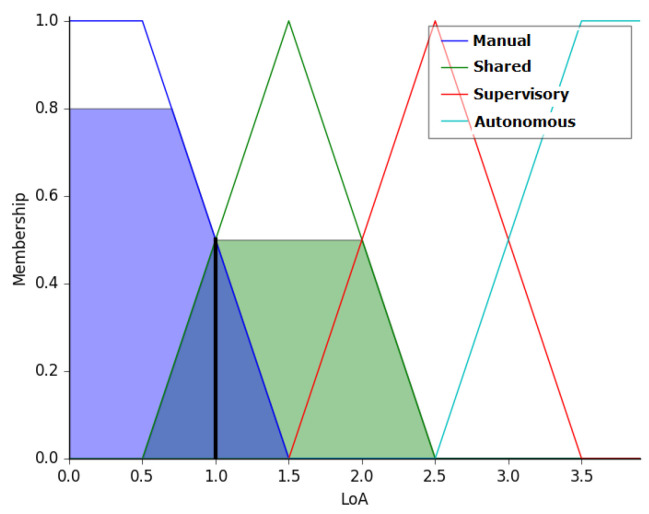
Output of the Fuzzy controller for the input vector (50, −1.5, −0.5 −0.3). The level of autonomy in the output is Manual Mode.

Reanalyzing this case, when the arm rotation measured by the MyoRMS value is corrected to −0.3, the input vector is modified to (50, −1.5, −0.5, −0.3). The output in this case is 1.00, which represents manual mode, as shown in Figure 15.

## 4. Experiments and Results

**Figure 16:**
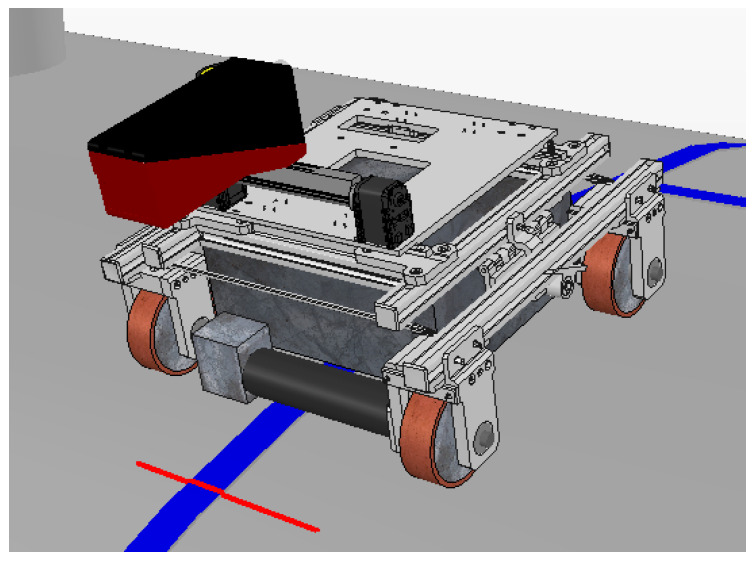
Autonomous Inspection Robot 1 (AIR-1) in the V-REP simulator.

The experiments were validated in a simulation environment using the Virtual Robot Experimentation Platform (V-REP) developed by Coppelia Robotics. This software implements a realistic physics system and total integration with ROS via topics. As ROS topics are standardized, all nodes and the fuzzy controller function similarly regardless of whether the system is simulated or is the real world and sensors. Figure 16 shows the virtual AIR-1 model along with its sensors. The red line represents the lasers emitted by the LRS36/6 virtual profile sensor.

**Figure 17:**
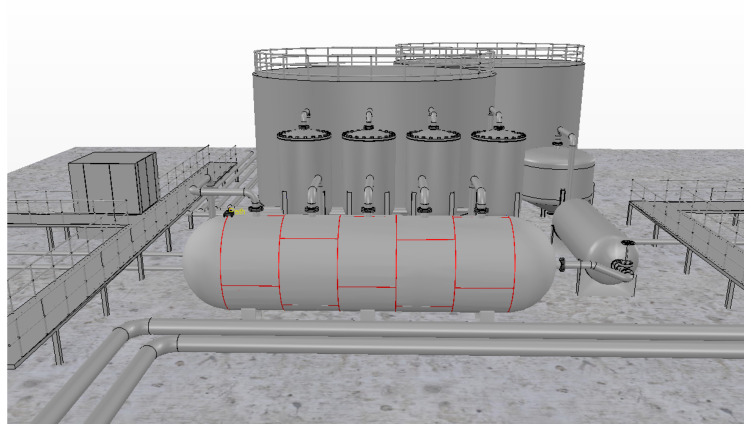
Scene used of a refinery with storage ranks and weld beads in the V-REP simulator.

In this simulation environment, a scene containing a refinery with storage tanks and real-world dimensions was used. Figure 17 shows this scene, with the cylindrical tank with weld beads (highlighted in red) being the object of interest.

### 4.1. Experiments with a Fixed Level of Autonomy

**Figure 18:**
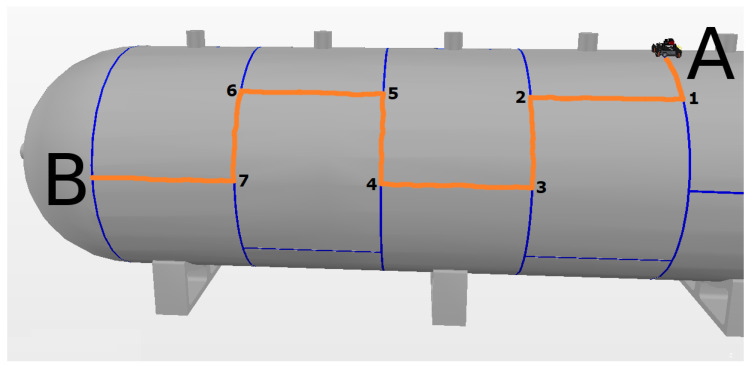
Navigation goal of the experiments. Point A is the initial position of the robot and point B is the final position. The optimal course is shown in orange and the turns are numerated.

First, the levels of autonomy were validated separately, which were set before the experiment and remained the same throughout the experiment. In these experiments, the operator had the goal of navigating through the storage tank with the robot as centralized as possible in the weld bead, from the starting point A to the final point B, taking 7 turns, as shown in Figure 18. The experiments were repeated five times with each autonomy level, and to measure the efficiency during the experiments, the time spent during the task and the average of the alignment error of the weld bead relative to the center of the robot were evaluated.

#### 4.1.1. Manual Mode Experiments

In this experiment, the operator controlled the robot via the industrial joystick together with the Myo armband IMU rotation angle measured to navigate through the tank while maintaining the robot in the weld bead line. The experiments performed in manual mode are presented in Table 5.

**Figure 19:**
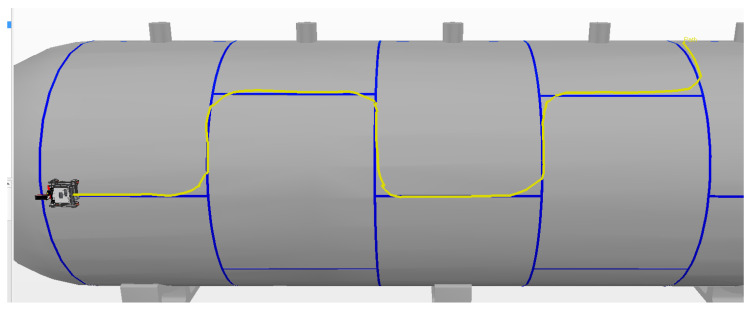
Manual Mode. The yellow line is the route traveled by the robot.

The best result in manual mode was achieved during the first experiment, which had a smaller alignment error of 12.8 cm. The route covered by this experiment is shown by the yellow line in Figure 19.

**Figure 20:**
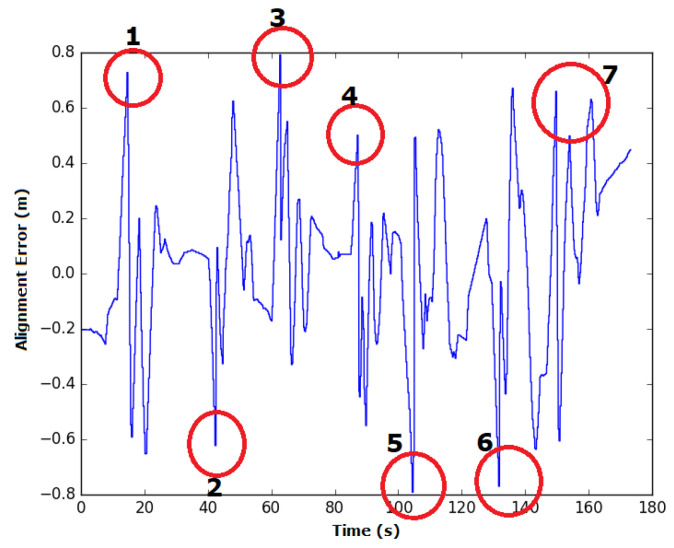
Variation in the alignment error of the robot during experiment 1 in manual mode. The spikes in the error occur during turns due to the robot’s topology and are circled in red, with a number representing the associated curve.

In manual mode, the time spent during each experiment was similar, with an average of 174.9 s and standard deviation of 4.5 s. The average alignment error was also similar between experiments, with an average of 14 cm and standard deviation of 0.8 cm. The instant error of this experiment is presented in Figure 20.

Some spikes in the instant error can be observed when the robot was taking/executing turns. Based on the way the robot moves and the positioning of the profile sensor on the robot, it is impossible to obtain zero error along the curves. In these sections, the weld bead moves away from the center of the robot until it reaches a point where changes in the weld bead occur, which will be detected for the next segment to be covered, inverting the signal of the alignment error. This error peaks at a positive value when the curve is to the left of the robot and has a high negative value when the curve is to its right. These points are highlighted in the figure and numbered according to the relative curve. However, between these peaks, the lack of skill on the operator’s part makes him/her incapable of reducing the alignment error to zero in manual mode.

#### 4.1.2. Shared Mode Experiments

The experiments in shared mode were conducted with a 50–50 relationship, which means that 50% of the final speed of the robot was input by the operator with the joystick and Myo armband and 50% was achieved with the autonomous mode of the robot. The results are presented in Table 6.

**Figure 21:**
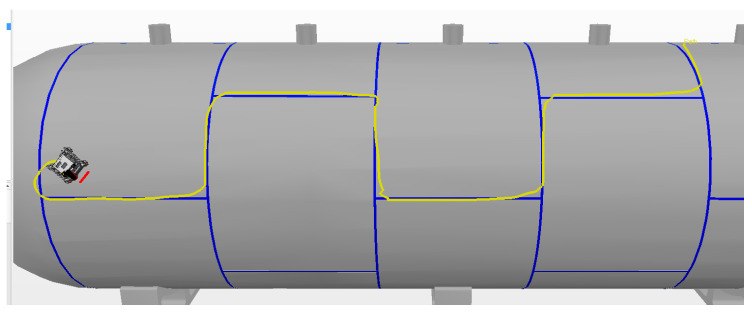
The experiment was performed in shared mode with 50% of the velocity controlled by the operator and 50% by the robot.

The best result in relation to the average alignment error was achieved in the third experiment with 9.5 cm. An improvement of approximately 25% in the alignment error was observed compared with the experiments in manual mode because the factor of the autonomous mode played a part in the robot velocities. The trajectory described by the robot covers more of the weld bead mainly on the straight segments of the tank, as shown in Figure 21.

**Figure 22:**
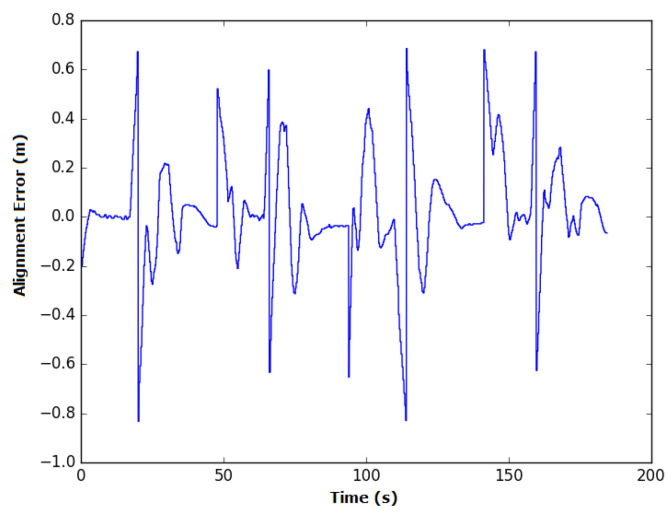
Variation in the alignment error during experiment 3 in Shared Mode.

In shared mode experiments, the elapsed time of the runs was longer, with an average of 185 s and standard deviation of approximately 8 s. This is because the speed output by the autonomous system, while steady, has a lower value than that of manual mode, which can be altered on the joystick. The first experiment required a longer time—i.e., 13 s above the average—than the other experiments likely due to the time required by the user to adapt to the new mode. On average, the alignment error showed a 4 cm improvement relative to manual mode. The instant error observed in the third experiment is presented in Figure 22.

It should be noted that the instant error is similar to that in manual mode with the spikes during/along the curves, but smoother in the straight sections, owing to the robot’s autonomous system sharing the speed control.

#### 4.1.3. Supervisory Mode Experiments

In this mode, the operator controls the linear speed and the autonomous system controls the angular speed of the robot, ensuring that it covers the weld bead. The experiments performed in the supervisory mode are presented in Table 7.

**Figure 23:**
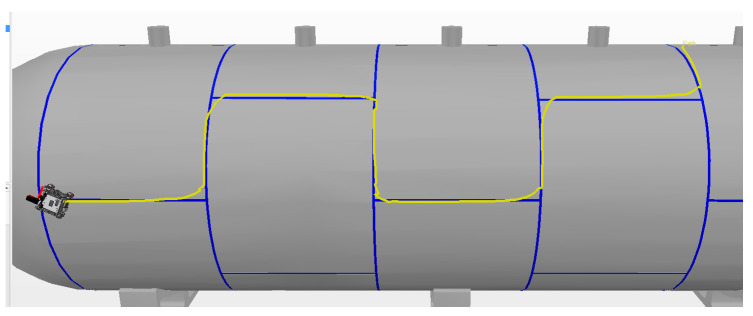
Experiment carried out in Supervisory Mode.

In this mode, the best result was achieved in the second experiment, with an average alignment error of 7.9 cm. The path of this experiment, as shown in Figure 23, demonstrates that the robot was almost always on the weld bead, and moved away from the correct line only along the curves.

**Figure 24:**
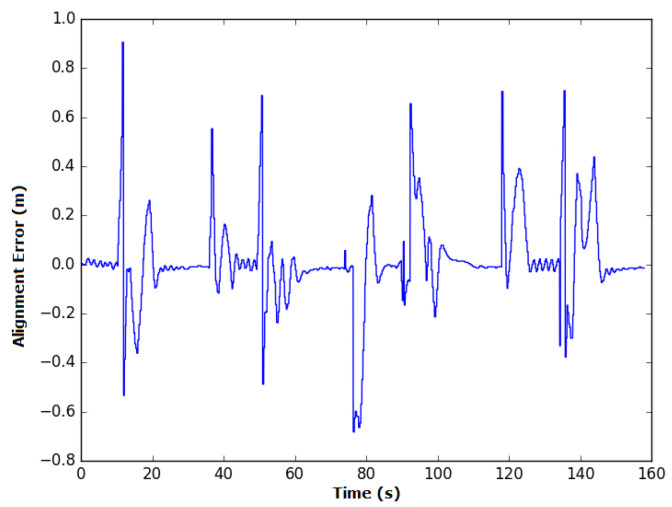
Variation in the alignment error of the robot in the course of experiment 2 in Supervisory Mode.

In the straight segments, while an improvement in the instant error was observed, as shown in Figure 24, the pattern of spikes in the errors during/along the curves was still present.

#### 4.1.4. Autonomous Mode Experiments

In this mode, the final position is set, and the robot finds the route along which to perform without the aid of the operator. The experiments in the autonomous mode showed similar results to those in the supervisory mode. This similarity was expected because the difference between these two modes lies in the linear velocity control. This difference is necessary in inspection tasks, as the operator needs to control the speed of the robot, pausing or halting in some segments, going slower or faster in others, and even going back to re-scan some segments. The results of the experiments performed in the autonomous mode are presented in Table 8.

These were as expected from the experiments with a lower standard deviation for an average alignment error of 2 cm. This illustrates the repeatability of the autonomous system when executing the same task. The average alignment error was 10.5 cm. The time elapsed in four of the five experiments was very similar, resulting in a smaller standard deviation among all the experiments with a fixed level of autonomy. However, due to an anomaly in the first experiment, which took an extra 6 s, the average was 157 s with a standard deviation of 2.5 s.

**Figure 25:**
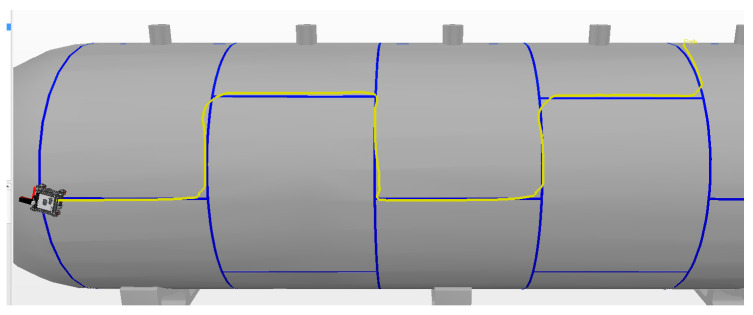
Experiment performed with Autonomous Mode.

Among all these experiments, the best result was obtained in the third experiment with an alignment error of 10.2 cm. The path of this experiment is illustrated in Figure 25.

The path shown is very similar to that in the supervisory mode; however, there is an improvement in the weld bead following the fourth curve, to the right. This curve presented some difficulties during the experiments and will be discussed in detail later.

**Figure 26:**
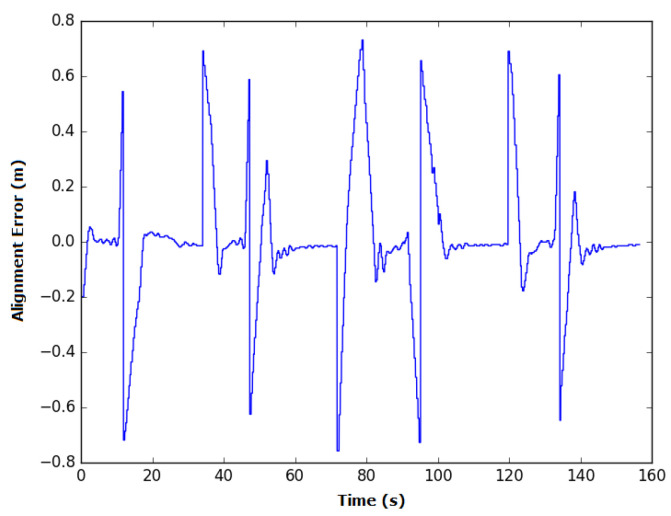
Variation of the alignment error during experiment 3 with Autonomous Mode.

The instant error during this experiment followed the previously observed pattern, with spikes on the curves and stability in the straight segments. The instant error is presented in Figure 26. The minimum alignment error with the autonomous mode is 2.5 mm in straight segments, as described by [52] in his work about the profile sensor and weld bead follower mode. However, the turns present in the tank generate spikes as high as 60 cm, bringing the average of the alignment error to about 10 cm. The theoretical minimum alignment error for this profile sensor could be 1 mm, which is the sensor’s resolution.

### 4.2. Experiments with Sliding Autonomy

To validate the proposed fuzzy controller, the same experiment with fixed autonomy levels was conducted with the controller, achieving a change in the autonomy during operation or with sliding autonomy. To demonstrate the above, five participants were invited to run the experiment only once. The profiles of the participants were: all male, in the age group of 20–31 years with experience in mobile robotics ranging from 6 months to 3 years; however, none of them had previous experience with the Myo armband or this model of joystick. Each participant performed the experiment only once to verify/evaluate the adaptability of the system to new users with different skill levels. The key information is whether the system is capable of adapting and correcting operator movements with different levels of expertise. If the participants had received training to enhance their skills before the experiments, it would be harder to verify the system’s adaptability. The protocol adopted for the experiments is listed below:

1. First, the participant and researcher were brought/accompanied to the computer, with the simulator and scene already open.
2. The goal of the experiment—i.e., going from point A to point B, covering as much as the weld bead as possible, and in as little time as possible—was presented orally to the participant.
3. The participant was asked to hold the joystick and wear the Myo armband and its controls were presented orally.
4. The participant was allowed a 15 min practice session with the same scene in manual mode.
5. At the end of the practice session, the scene was reset to the initial position and the fuzzy controller was activated to start the experiment.

The results are presented in Table 9.

The first column of Table 9 shows the participant number. The second column is the elapsed time during the experiment, and the subsequent columns present the percentages of the elapsed time that the fuzzy system took to select the autonomy levels in the order manual, shared, supervisory, and autonomous. The next column shows the arithmetic mean of the autonomy level output by the controller. The lower this value is, the higher the level of robotic control provided by the system to the operator, indicating greater experience and skill level. The second to last column shows the number of transitions between the autonomy levels and the last column presents the average alignment error during the experiments.

**Figure 27:**
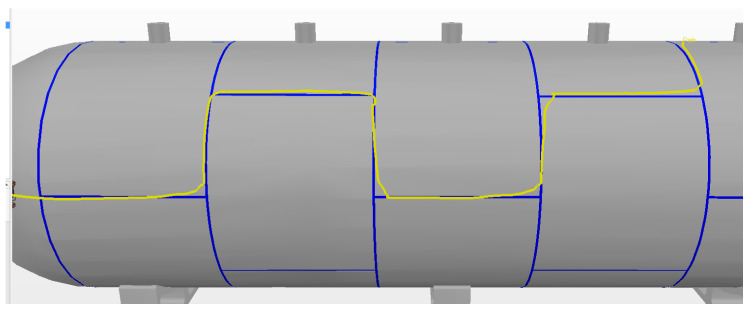
Sliding Autonomy experiment.

As an example, the fifth experiment is detailed here. Figure 27 shows the path covered.

**Figure 28:**
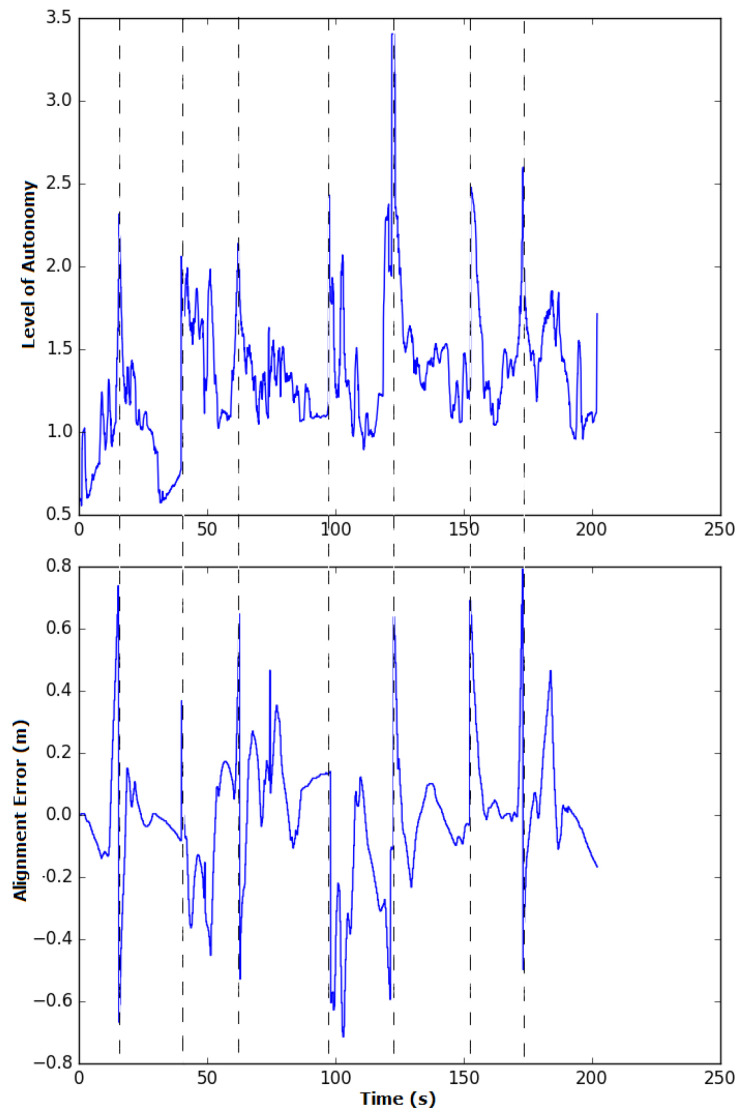
Autonomy and alignment error during the sliding autonomy experiment. The panel above shows the output of the fuzzy controller during the experiment, representing the level of autonomy. The panel below shows the alignment error. The dashed lines indicate the instances when there were spikes in the errors and in the level of autonomy.

It is perceived that along some curves—i.e., the second curve, to the left, and fourth curve, to the right—the path shows sudden angles that were not found in the previous experiments with fixed levels of autonomy. This is because the autonomy is changed online and, at these curve points, there are also spikes in the alignment error, resulting in the controller increasing its autonomy by pursuing better weld bead tracking. These values can be corroborated in Figure 28, with the output of the fuzzy controller shown above and the alignment error recorded during the experiment shown below.

Initially, the system had a low level of autonomy, indicating that the participant was moving the robot correctly, mainly because in this part the robot was already aligned and only had to follow a straight path until the first curve. At the curves, there was an increase in the level of autonomy, either by the struggle in maintaining the robot in the weld bead or by the robot’s topology, which prevented perfect weld bead tracking along the curves. At some points, there were also spikes in the autonomy level, which corresponded to the spikes in the alignment error identified in the curves, as shown by the dashed lines in Figure 28. It can also be observed from this figure that the level of autonomy was mainly in the 1–2 range, as evidenced by the table, which shows that the selected autonomy level was shared during 81.73% of the time. Between 100 and 150 s, the system was in the autonomous mode for 0.65% of the total time elapsed, indicating that during a curve, the participant was unable to navigate the robot correctly and the autonomous system took over to maintain proper functioning.

### 4.3. Results and Discussion

To ensure better analysis, the average of the experiments was performed, while rejecting the best and worst experiments in each case. Table 10 presents these results.

Considering only the experiments with a fixed level of autonomy, it can be seen that the worst result in terms of the average alignment error was obtained in manual mode. The result in shared mode was 28% better than that in manual mode, with a 6% increase in time elapsed to perform the task. The extra time was due to the orientation corrections performed by the autonomous system.

**Figure 29:**
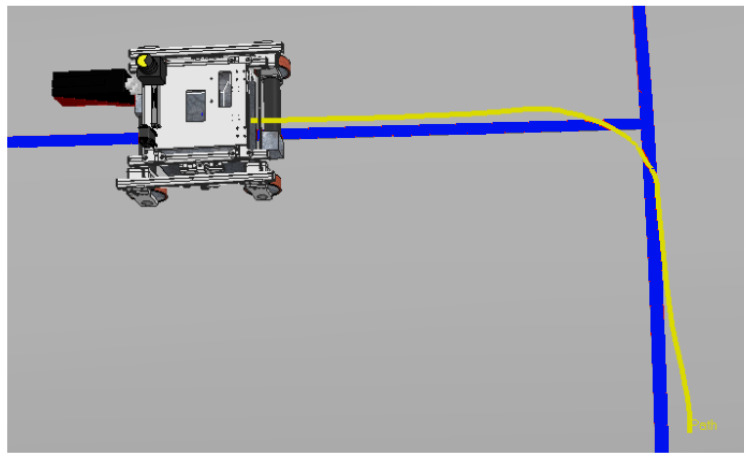
Path described by AIR1 during a curve.

Comparing Figure 20 and Figure 26, which represent the instant error in manual and autonomous modes, respectively, the difficulties of manually maintaining the robot in the weld bead along the straight segments can be observed. In the figures mentioned above, this corresponds to the moments/instances where the error is close to zero in the autonomous and supervisory modes—moments that were not observed while in manual or shared modes. Nonetheless, in all the modes, spikes can be observed in the instant error figures at the curves owing to the previously mentioned topology of the robot that is unable to follow 100% of the weld bead at the turns and changes of segments. Figure 29 shows the path described by the robot during a curve. The intersection of two weld beads makes a right angle, but the robot moves forward and turns at the same time, covering a curved, almost circular path during the curve. The discrepancy of the robot’s path and the weld bead line generates the spikes found in the figures, showing the alignment error during the experiments. The robot’s topology would have to change to minimize this since AIR1 cannot rotate around itself while not moving forward or backward. To achieve the right angle of the weld beads intersection, the robot would have to rotate around the center of the profile sensor, so the profile sensor would have to be repositioned. In a real-life situation of inspecting a weld bead line, the spikes in alignment error at curves are not crucial. The inspection task can be divided into several straight segments inspection tasks, and the curves are only used to reach new weld bead lines.

**Figure 30:**
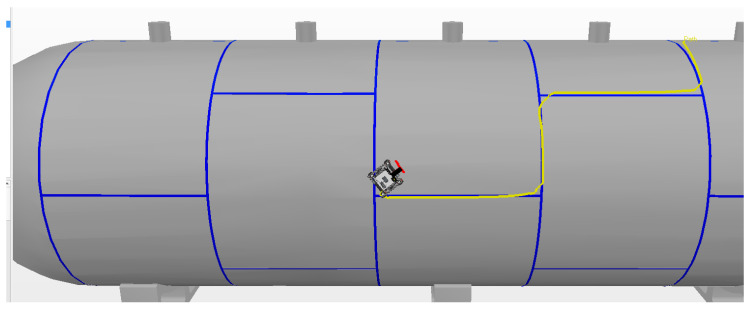
Emphasis on a curve where difficulties were encountered.

In supervisory and autonomous modes, the shortest times were observed with very similar results, with a slight advantage in the case of the autonomous mode. However, the best performance in terms of the accumulated alignment error during the task was observed in the supervisory mode, as the robot was aided by the operator, mainly along the curves, in making turns that were smoother and closer to the weld bead. In autonomous mode, struggles at the curves and even lost sight of the weld bead in some moments/instances due to the tank curvature and gravitational force. The curve routines must be modified for each situation. A frequently observed situation was encountered in curve 4, as shown in Figure 30.

At this moment, the robot had to perform a turn to the right while climbing. The autonomous system sometimes failed to perform this task, overturning and losing sight of the weld bead, and virtually getting lost in the tank, thus requiring human aid to find the weld bead again. In the other modes, this problem was not observed because the operator could manually control the curve and instinctively deliver the correct velocities. Through these experiments, the benefits of both the autonomous system and operator can be seen—i.e., the ability of the autonomous system to maintain the track of the weld bead during the straight segments and the adaptability of the operator to control the velocities of different types of curves.

Considering the sliding autonomy experiments with the fuzzy controller, the task required the longest time as compared with all the experiments. This increased time was due to a large variation in the preference and skill set of the participants. Based on the alignment error, the performance in the sliding autonomy mode was very similar to that in the supervisory mode, which showed the best performance in terms of the overall error.

Analyzing Table 9, a large variation between the skill and experience of the participants can be observed. The average output of the fuzzy, presented in the seventh column as “A. Aut.”, indicates how skilled the fuzzy controller thinks the operator is, and the smaller this value is, the more skilled the operator. During the experiment with the first participant, the controller output the manual mode for 43% of the experiment duration, indicating that this participant had a good grasp of the controls of the robot. In the third experiment, while only 5% of the experiment duration was in manual mode, the complete experiment was performed in the shortest time, indicating a hastier operator who did not care much about the weld bead covering. Only the fifth participant experienced a moment in the autonomous mode, for only 0.65% of the task completion time, over critical curve 4 where difficult climbing was required. While the number of transitions between the modes was not a relevant indicator/measure of the skill of the operator or alignment errors, the high number of transitions validates the developed controller, as a high number of transitions would not have occurred if the operator had to control the robot and change the level of autonomy during the operation.

The most important observation in this table is that even with a long elapsed time of the task variation of approximately 15 s between participants or a high average output of the controller of approximately 0.47 points, all experiments showed a very low alignment error that was very similar among the participants. This finding is the most important aspect of this work, as it validates the correction and adjustment mechanism implemented by the fuzzy controller for different operators with different levels of experience.

## 5. Conclusions

The goal of this study was to develop a human–robot interface based on sensor fusion combined with intelligent strategies in a climbing robot for the inspection of storage tanks and pressure vessels.

The developed interface was needed so that different operators with different skill and experience levels could control the robot during inspection tasks with a high weld bead cover.

While a fully autonomous mode is possible, the task of inspecting the tanks is still highly dependent on a human operator operating and visualizing the phased-array ultrasonic sensors, so the path and the velocity described by the robot during this task can vary greatly. An operator may need to go back in a segment and redo the inspection or focus on a specific area of the tank. A fully autonomous mode can only be achieved once the inspection task with the ultrasonic sensors becomes fully autonomous. An analysis of specialized literature in the robotics context has revealed four levels of autonomy, namely the manual, shared, supervisory, and autonomous modes. These modes were developed and implemented via the fusion of sensors. An industrial joystick that works with radio frequency was designed, and a driver was developed to control the robot in combination with an um electromyographic armband that can distinguish between hand signals, rotation angles, and the level of stiffness of an arm. These two inputs were fused to generate more fluid and smooth movements of the robot. Some of the implemented levels of autonomy used the weld bead follower measured by a profile sensor and a controller presented in the study in [52]. After all the modes were separately implemented, a fuzzy controller was developed to obtain the best level of autonomy during the operation time, allowing the robot to adapt to the current operator. The inputs of this controller include the weld position relative to the center of the robot measured by the profile sensor, joystick inputs, and armband inputs.

Validation of the system was performed through virtual experiments using the V-REP simulator. In the virtual environment, a scene containing a storage tank with weld beads and an AIR-1 inspection robot were used to cover a route simulating an inspection task. First, the experiments were performed with fixed levels of autonomy considering metrics such as the execution time of the task and average alignment error of the weld bead. In the other experiments performed with sliding autonomy, five participants with different skill levels controlled the robot through the same course, while trying to keep the robot as close as possible to the weld bead to the best of their abilities. During the sliding autonomy experiments, despite all participants having different skill levels, it was verified that the alignment error of the weld bead was very similar, thus proving that the system could adapt to different users.

Real-life experiments are on the horizon to verify the validity of the proposed approach. The system is expected to achieve results similar to the simulated experiments. The robot’s low speed and smooth controlling of the joystick and armband suggest that the interface’s feedback delays would not be an issue.

## Figures and Tables

**Table 1:** Sliding Autonomy in different fields.

Scope	Levels	Offline	Online
Mobile Robots	2-4	[15] [16] [12] [14]	-
Aviation and Space	3-4	[17] [18] [19]	[20] [21] [22]
Self-Driving Cars	3-6	[23]	[24] [25]
Search and Rescue	2-3	[26]	[27]
Military	2-3	[28]	[29]

**Table 2:** Rules for angular velocity and weld position.

JoyAngular|WeldPos	LH	LL	CT	RL	RH
LH	MN	SH	SP	AT	AT
LL	SH	MN	SH	SP	AT
CT	SP	SH	MN	SH	SP
RL	AT	SP	SH	MN	SH
RH	AT	AT	SP	SH	MN

**Table 3:** Rules for roll angle of the arm and weld position.

MyoRoll|WeldPos	LH	LL	CT	RL	RH
NH	MN	SH	SP	AT	AT
NL	SH	MN	SH	SP	AT
ZR	SP	SH	MN	SH	SP
CL	AT	SP	SH	MN	SH
CH	AT	AT	SP	SH	MN

**Table 4:** Rules for MyoRMS and Level of Autonomy.

MyoRMS	Level of Autonomy
MH	Supervisory
HG	Autonomous

**Table 5:** Manual Mode Experiments.

Experiment	Elapsed Time (s)	Average of the Alignment Error (m)
1	173.26	0.1283
2	173.27	0.1484
3	169.53	0.1349
4	176.73	0.1437
5	181.48	0.1460
Average	174.85	0.1403
Standard deviation	4.49	0.0084

**Table 6:** Shared Mode Experiments.

Experiment	Elapsed Time (s)	Average of the Alignment Error (m)
1	198.06	0.0992
2	176.91	0.1051
3	184.46	0.0955
4	180.81	0.1014
5	185.97	0.1073
Average	185.24	0.1017
Standard deviation	7.98	0.0047

**Table 7:** Supervisory Mode Experiments.

Experiment	Elapsed Time (s)	Average of the Alignment Error (m)
1	160.25	0.1078
2	158.02	0.0797
3	159.01	0.0954
4	158.85	0.0927
5	156.99	0.0883
Average	158.62	0.0928
Standard deviation	1.21	0.0103

**Table 8:** Autonomous Mode Experiments.

Experiment	Elapsed Time (s)	Average of the Alignment Error (m)
1	162.29	0.1067
2	156.47	0.1066
3	156.59	0.1021
4	156.21	0.1029
5	157.44	0.1068
Average	157.80	0.1050
Standard deviation	2.55	0.0024

**Table 9:** Sliding Autonomy Experiments.

E.	Time (s)	% Man.	% Shar.	% Sup.	% Aut.	A. Aut.	Tr.	Error
1	204.51	43.01	55.45	1.54	0.00	1.06	108	0.1032
2	195.01	13.55	83.45	3.00	0.00	1.29	60	0.1019
3	189.76	5.18	83.98	10.84	0.00	1.53	37	0.0907
4	215.25	10.32	76.32	13.36	0.00	1.51	66	0.1029
5	202.00	13.36	81.73	4.26	0.65	1.34	55	0.1068

**Table 10:** Summary of the Experiment Results.

Level of Autonomy	Average of the Alignment Error (m)	Average of Time (s)
Supervisory	0.0922	158.28
Sliding	0.0982	204.09
Shared	0.1019	185.26
Autonomous	0.1054	158.32
Manual	0.1415	175.91
